# Differences between Spinocerebellar Ataxias and Multiple System Atrophy-Cerebellar Type on Proton Magnetic Resonance Spectroscopy

**DOI:** 10.1371/journal.pone.0047925

**Published:** 2012-10-31

**Authors:** Jiing-Feng Lirng, Po-Shan Wang, Hung-Chieh Chen, Bing-Wen Soong, Wan Yuo Guo, Hsiu-Mei Wu, Cheng-Yen Chang

**Affiliations:** 1 National Yang-Ming University School of Medicine, Taipei, Taiwan; 2 Department of Radiology, Taipei Veterans General Hospital, Taipei, Taiwan; 3 Department of Neurology, National Yang-Ming University School of Medicine, Taipei, Taiwan; 4 Department of Medicine, Municipal Gandau Hospital, Taipei, Taiwan; 5 Department of Radiology, Taichung Veterans General Hospital, Taipei, Taiwan; 6 Department of Neurology, Taipei Veterans General Hospital, Taipei, Taiwan; University of Edinburgh, United Kingdom

## Abstract

**Purpose:**

A broad spectrum of diseases can manifest cerebellar ataxia. In this study, we investigated whether proton magnetic resonance spectroscopy (MRS) may help differentiate spinocerebellar ataxias (SCA) from multiple systemic atrophy- cerebellar type (MSA-C).

**Material and Methods:**

This prospective study recruited 156 patients with ataxia, including spinocerebellar ataxia (SCA) types 1, 2, 3, 6 and 17 (N = 94) and MSA-C (N = 62), and 44 healthy controls. Single voxel proton MRS in the cerebellar hemispheres and vermis were measured. The differences were evaluated using nonparametric statistic tests.

**Results:**

When compared with healthy controls, the cerebellar and vermis NAA/Cr and NAA/Cho were lower in all patients*(p<0.002)*. The Cho/Cr was lower in SCA2 and MSA-C *(p<0.0005)*. The NAA/Cr and Cho/Cr were lower in MSA-C or SCA2 comparing with SCA3 or SCA6. The MRS features of SCA1 were in between *(p<0.018)*. The cerebellar NAA/Cho was lower in SCA2 than SCA1, SCA3 or SCA6 *(p<0.04)*. The cerebellar NAA/Cho in MSA-C was lower than SCA3 *(p<0.0005)*. In the early stages of diseases (SARA score<10), significant lower NAA/Cr and NAA/Cho in SCA2, SCA3, SCA6 or MSA-C were observed comparing with healthy controls *(p<0.017)*. The Cho/Cr was lower in MSA-C or SCA2 (*p<0.0005*). Patients with MSA-C and SCA2 had lower NAA/Cr and Cho/Cr than SCA3 or SCA6 *(p<0.016)*.

**Conclusion:**

By using MRS, significantly lower NAA/Cr, Cho/Cr and NAA/Cho in the cerebellar hemispheres and vermis were found in patients with ataxia (SCAs and MSA-C). Rapid neuronal degeneration and impairment of membrane activities were observed more often in patients with MSA-C than those with SCA, even in early stages. MRS could also help distinguish between SCA2 and other subtypes of SCAs. MRS ratios may be of use as biomarkers in early stages of disease and should be further assessed in a longitudinal study.

## Introduction

Cerebellar ataxias can be categorized into two groups: hereditary or sporadic [1]. Sporadic cerebellar degeneration may be the result of intoxication, endocrine disorders or idiopathic causes, such as multiple system atrophy-cerebellar type (MSA-C). Hereditary ataxias have different modes of inheritance with mutations in scores of genes. Spinocerebellar ataxia (SCA) represents the most common autosomal dominant cerebellar ataxia and so far 36 distinct chromosomal loci have been identified. They share similar manifestations, including ataxia, pyramidal and extrapyramidal signs. The diagnosis of MSA-C can only be reached when clinical and laboratory criteria are met in accordance with the international consensus [2]. Patients with MSA-C usually have a rapid clinical progression with a mean survival of 6–9 years. An accurate diagnosis not only foretells the prognosis, makes comprehensive genetic counseling possible, but also helps design the best clinical management.

Atrophy and signal intensity changes in the cerebellum and brain stem can be observed in both SCA and MSA-C using magnetic resonance images. Although some characteristic features, such as abnormal signal intensities in the middle cerebellar peduncle, the “hot cross bun” sign and putaminal slit, may be observed in patients with MSA [3], they usually are only discernible in the late stages of the disease.

Creatine (Cr) measured in magnetic resonance spectroscopy (MRS) is related to cell energy pathways and reflects the energy potential available in the brain tissue. Its concentration in normal brain remains very high (7.49±0.12 mmol/kg) and stable and can be used as a reference for comparisons. N-acetylaspartate (NAA) is believed to be neuronal and axonal origin in adult brain [4], [5] and is a marker for neuronal integrity, density or volume [6], [7]. A reduction in NAA resonance has been observed in various conditions associated with neuronal loss and/or axonal injury [8]–[12]. Choline (Cho) resonance reflects total Cho stores, derived from glycerophosphocholine and phosphocholine [5]. Loss of Cho reflects a reduction in the production of cell membranes, the neurotransmitter acetylcholine and the precursor of acetylcholine, phosphatidylcholine.

Previously, changes in MRS in patients with SCA or MSA-C have been reported. [13]–[17]. However, most of the reports only focused on limited subtype of SCA or only described the differences between patients and healthy controls.

The purpose of this study was to investigate whether noninvasive MRS may help differentiate between SCAs and MSA-C and between SCA subtypes on a cross-sectional basis.

## Materials and Methods

### Patients and Controls

This study was approved by the Institutional Review Board of Taipei Veterans General Hospital, Taipei, Taiwan. From March 2004 to March 2010, after written informed consent was signed, a total of 94 patients with SCA and 62 patients with MSA-C were recruited into this study. Four patients were molecularly identified to have SCA1, 16 SCA2, 58 SCA3, 10 SCA6 and 6 SCA17. Sixty-two patients met the criteria for MSA-C. The age and disease duration were used as co-variances for MRS parameter analyses. All patients periodically underwent neurological examination, including “Scale for the Assessment and Rating of Ataxia” (SARA) [18], [19]and neuroimaging studies with MRS. Forty-four healthy individuals without any history of neurological diseases served as controls. The basal ganglion MRS features were validated, according to normal data proposed by Brain Ross and Else Rubaek Danielsen [20]. The demographic features are listed in Table 1.

**Table 1:** Demographic features of the subjects.

	No. of patients	CAG repeat length	Disease Duration # (years)	Age at study # (years)
SCA1	4	43.8±4.4	12.3±12.9	56.0±2.3
SCA2	16	42.9±6.2	6.5±4.0	44.9±16.9
SCA3	58	73.2±3.9	8.9±6.2	50.0±12.7
SCA6	10	23.6±1.1	8.3±7.7	56.7±11.3*
SCA17	6	44.5±2.2	3.0±3.2§	52.0±15.5
MSA-C	62	–	5.3±3. 6§§	62. 6±7.1**
Healthy controls	44	–	–	51.1±17.9

# Kruskal-Wallis test, *p*<0.05.

§ Patients with SCA2 or SCA3 had a longer disease duration than those with SCA17, *p*<0.05.

§§ Patients with SCA3 had a longer disease duration than those with MSA-C.

* Patients with SCA2 or SCA3 were younger than those with SCA6.

** Patients with SCA were younger than those with MSA-C.

### Image and spectroscopic acquisition

Brain MRI and MRS were performed using a 1.5-T system (Signa EXCITE, GE Medical Systems, Milwaukee, WI). The MRI protocol consisted of an axial T1-weighted three-dimensional fast-spoiled gradient recalled acquisition in steady state images (TR 8.58 msec, TE 3.62 msec, inversion time [TI] 400 msec, voxel resolution 0.75×0.75×1.5 mm^3^) and an axial T2 fast spin-echo sequence [TR 4000 msec, TE 256.5 msec, voxel resolution 348×512].

After MR imaging, proton MRS was recorded in cerebellar hemispheres and cerebellar vermis by using single-voxel stimulated echo acquisition mode sequence (3000/15/13.7/96 [TR/TE/mixing time/excitations], spectral width = 2500 Hz, number of points = 2048, voxel resolution = 2 cm×2 cm×2 cm). The voxel of interest (VOI) in each subject was placed in a uniform manner by the same investigator (JFL). Care was taken to avoid cerebrospinal fluid spaces within the VOIs. The peak areas for N-acetyl aspartate (NAA) at 2.02 parts per million (ppm), Creatine (Cr) at 3.03 ppm, and Choline (Cho) at 3.22 ppm were measured using the Functool provided by MR company (GE XVi, Milwaukee, WI). Peak integral values were expressed relative to the Cr peak. Metabolite intensity ratios were automatically calculated at the end of each single voxel acquisition including NAA/Cr and Cho/Cr. The NAA/Cho ratio was also calculated for comparison. In order to ensure high quality, MRS results with full width at half maximum (FWHM)>6 Hz were disqualified from the MRS analyses.

### Statistical Analyses

Comparisons of the age at examination and disease duration between the healthy controls and patient groups were performed using nonparametric statistics (Kruskal-Wallis H test) since some of the sample sizes were small and the assumption of a Gaussian distribution was not appropriate.

Comparison of the metabolic parameters on MRS (NAA/Cr, Cho/Cr and NAA/Cho in the cerebellar hemispheres, NAA/Cr, Cho/Cr and NAA/Cho in the vermis) between patient groups and healthy controls, between sub-types of SCA and MSA-C and between sub-types of SCA were all performed using the non-parametric Mann-Whitney U-test due to non-Gaussian distribution of the MRS parameters. Differences were considered significant at *p*<0.05. Data are given as mean ± standard deviation.

Metabolic ratios on the MRS, clinical ataxia scores (SARA) and disease duration in individual patients were correlated using Spearman's rank test. Differences were considered significant at *p*<0.05.

## Results

Overall, two NAA/Cr and two Cho/Cr in the vermis from healthy controls, two NAA/Cr and one Cho/Cr in the cerebellar hemisheres, 14 NAA/Cr and 15 Cho/Cr in the vermis from patients with SCA, and 8 NAA/Cr and 6 Cho/Cr in the cerebellar hemispheres, 8 NAA/Cr and 7 Cho/Cr in the vermis from patients with MSA-C were disqualified.

Altogether, 88 NAA/Cr, Cho/Cr and NAA/Cho in the cerebellar hemispheres and 42 NAA/Cr, Cho/Cr and NAA/Cho in the vermis from healthy controls, 8 NAA/Cr, Cho/Cr and NAA/Cho in the cerebellar hemispheres and 3 NAA/Cr, Cho/Cr and NAA/Cho in the vermis from patients with SCA1, 31 NAA/Cr, 32 Cho/Cr and 31 NAA/Cho in the cerebellar hemispheres and 14 NAA/Cr, Cho/Cr and NAA/Cho in the vermis from patients with SCA2, 115 NAA/Cr, Cho/Cr and NAA/Cho in the cerebellar hemispheres and 48 NAA/Cr, 47 Cho/Cr and 47 NAA/Cho in the vermis from patients with SCA3, 20 NAA/Cr, Cho/Cr and NAA/Cho in the cerebellar hemispheres and 10 NAA/Cr, Cho/Cr and NAA/Cho in the vermis from patients with SCA6, 12 NAA/Cr, Cho/Cr and NAA/Cho in the cerebellar hemispheres and 5 NAA/Cr, Cho/Cr and NAA/Cho in the vermis from patients with SCA17, 116 NAA/Cr, 118 Cho/Cr and 114 NAA/Cho in the cerebellar hemispheres and 54 NAA/Cr, 55 Cho/Cr and 54 NAA/Cho in the vermis from patients with MSA-C were included in the statistic analyses.

### Copmparison with healthy controls

#### Patients with MSA-C

**Figure 1:**
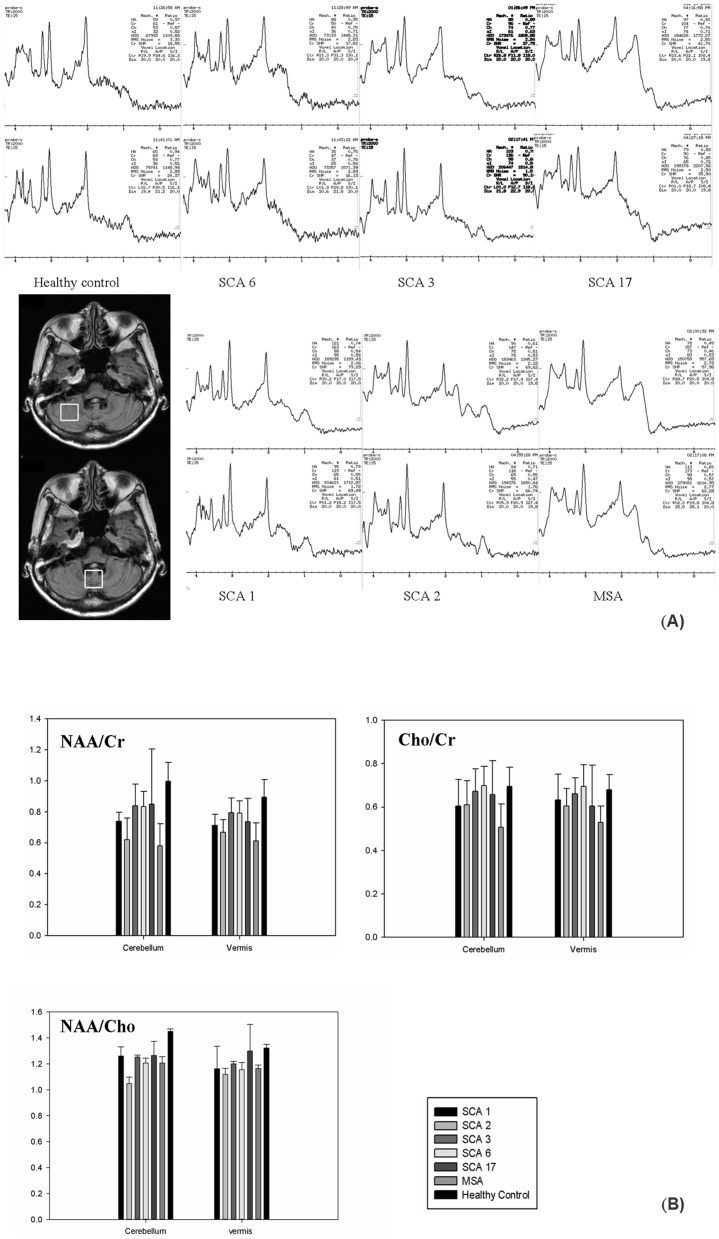
Representative MR spectra. (A) The MR spectroscopy in the cerebellar hemispheres (upper row) and vermis (lower row) in healthy controls and patients with SCA6, SCA3, SCA17, SCA1, SCA2 or MSA-C. (B) Group comparisons of NAA/Cr, Cho/Cr and NAA/Cho between the patients and controls.

By Mann-Whitney test, the NAA/Cr, Cho/Cr and NAA/Cho in the cerebellar hemispheres and vermis were significantly lower (*p*<0.0005) in patients with MSA-C. (Fig. 1 and Table 2)

**Table 2:** The MR spectroscopy features of the study participants.

	Cerebellar hemispheres			Cerebellar vermis		
	NAA/Cr	Cho/Cr	NAA/Cho	NAA/Cr	Cho/Cr	NAA/Cho
SCA1	0.74+/−0.06*	0.61+/−0.12*	1.26+/−0.2*	0.71+/−0.07*	0.63+/−0.12	1.16+/−0.3
SCA2	0.62+/−0.14*	0.61+/−0.11*	1.05+/−0.28*	0.67+/−0.08*	0.60+/−0.08*	1.12+/−0.17*
SCA3	0.84+/−0.14*	0.67+/−0.1	1.25+/−0.16*	0.79+/−0.1*	0.66+/−0.07	1.20+/−0.13*
SCA6	0.83+/−0.1*	0.70+/−0.09	1.21+/−0.17*	0.79+/−0.08*	0.70+/−0.1	1.16+/−0.18*
SCA17	0.85+/−0.36*	0.66+/−0.16	1.27+/−0.37	0.74+/−0.15*	0.60+/−0.19	1.30+/−0.46
MSA−C	0.58+/−0.14*	0.51+/−0.11*	1.20+/−0.53*	0.61+/−0.12*	0.53+/−0.08*	1.16+/−0.21*
Healthy controls	2.00+/−0.12	0.70+/−0.09	1.45+/−0.2	0.90+/−0.11	0.68+/−0.07	1.32+/−0.18

* Mann-Whitney test, *p*<0.05, compared with the healthy controls.

#### Patients with SCA

By Mann-Whitney test, the NAA/Cr in the cerebellar hemispheres and vermis were significantly lower in all subtypes of SCA (*p*<0.0005∼0.04). (Fig. 1 and Tab. 2)

The Cho/Cr in the cerebellar hemispheres and vermis were lower (*p*<0.0005, *p* = 0.001, respectively) in patients with SCA2, but only the Cho/Cr in the cerebellar hemispheres was lower (*p* = 0.022) in patients with SCA1.

The NAA/Cho in the cerebellar hemispheres and vermis was lower (*p*<0.0005∼0.007) in patients with SCA2, SCA3 and SCA6. In SCA1, only the NAA/Cho in the cerebellar hemispheres was lower *(p = 0.024)*.

#### Comparison between SCA and MSA-C

The NAA/Cr in the cerebellar hemispheres and vermis were lower in patients with MSA-C than those with SCA3 or SCA6 (*p*<0.0005, *p*<0.0005, respectively) and those with SCA1 or SCA 17. (*p*<0.0005, *p* = 0.004, respectively). (Table 2) There was no significant difference in the NAA/Cr in either cerebellar hemispheres or vermis between patients with SCA2 and those with MSA-C (*p* = 0.114, *p* = 0.085, respectively) (Table 2)

The Cho/Cr in the cerebellar hemispheres and vermis were lower (*p*<0.0005, *p* = 0.003, respectively) in patients with MSA-C when compared with those with SCA2, SCA3 (*p*<0.0005, *p*<0.0005, respectively), or SCA6 (*p*<0.0005, *p*<0.0005, respectively). The Cho/Cr in the cerebellar hemispheres in patients with MSA-C was lower than that of the patients with SCA1 or SCA 17. (*p* = 0.032, *p* = 0.001, respectively).

The NAA/Cho in the cerebellar hemispheres in patients with MSA-C was lower than those with SCA3 (*p<0.0005*).

#### Comparison between subtypes of SCA

The NAA/Cr in the cerebellar hemispheres and vermis was lower in patients with SCA2 when compared with those with SCA3 or SCA6 (*p*<0.0005∼0.001). The NAA/Cr in the cerebellar hemispheres was significantly lower in patients with SCA1 than those with SCA3 (*p* = 0.012) or SCA6 (*p* = 0.007) but higher than those with SCA2 (*p* = 0.002). In patients with SCA 17, the NAA/Cr in the cerebellar hemispheres was higher than those with SCA2 (*p* = 0.018). No difference was found between patients with SCA1, SCA3 or SCA6.

Patients with SCA2 had a lower Cho/Cr in the cerebellar hemispheres and vermis than those with SCA3 (*p* = 0.003; *p* = 0.008, respectively) or SCA6 (*p* = 0.004, *p* = 0.031, respectively). In patients with SCA1, the Cho/Cr in the cerebellar hemisphere was also significantly lower than those with SCA6 (*p* = 0.028) or SCA3 (*p* = 0.06). In patients with SCA 17, there was no significant difference in Cho/Cr, when compared with those with SCA1, SCA2, SCA3 or SCA6.

Patients with SCA2 had a lower NAA/Cho in the cerebellar hemispheres than those with SCA1 (*p = 0.04*), SCA3 (*p<0.0005*) or SCA6 (*p = 0.026*). There was no significant difference in NAA/Cho between patients with SCA1, SCA3, SCA6 or SCA17.

#### In the early stage of the diseases

In this study, 43 patients were in the early stage of the diseases, as defined by a SARA score below 10 unit points, including 6 patients with SCA2, 20 with SCA3, 4 with SCA6, 1 with SCA 17 and 12 with MSA-C. (Table 3)

**Table 3:** The MR spectroscopy features in early stage of diseases.

	Cerebellar hemispheres			Cerebellar vermis		
	NAA/Cr	Cho/Cr	NAA/Cho	NAA/Cr	Cho/Cr	NAA/Cho
SCA2	0.7+/−0.1*	0.6+/−0.1*	1.2+/−0.2*	0.7+/−0.1*	0.6+/−0.1*	1.2+/−0.2
SCA3	0.9+/−0.1*	0.7+/−0.1	1.3+/−0.1*	0.8+/−0.1*	0.7+/−0.1	1.2+/−0.1*
SCA6	0.9+/−0.1*	0.7+/−0.1	1.2+/−0.2*	0.8+/−0.1*	0.8+/−0.1*	1.0+/−0.1*
MSA−C	0.7+/−0.2*	0.6+/−0.1*	1.1+/−0.3*	0.7+/−0.1*	0.6+/−0.1*	1.2+/−0.1*
Healthy controls	1.0+/−0.1	0.7+/−0.1	1.5+/−0.2	0.9+/−0.1	0.7+/−0.1	1.3+/−0.2

* Mann-Whitney test, *p*<0.05, compared with the healthy controls.

### Patients with MSA-C

Significantly lower NAA/Cr, Cho/Cr and NAA/Cho in the cerebellar hemispheres and vermis were found in patients with MSA-C, according to Mann-Whitney U-test (*p<0.0005∼0.002*). (Table 4)

**Table 4:** The *p*-values derived from statistical analyses in early stage of diseases when SARA scores were below 10 unit points.

	Cerebellar hemispheres			Cerebellar vermis		
	NAA/Cr	Cho/Cr	NAA/Cho	NAA/Cr	Cho/Cr	NAA/Cho
SCA2	<0.0005	<0.0005	0.002	<0.0005	<0.0005	0.077
SCA3	<0.0005	0.147	<0.0005	0.002	0.457	0.007
SCA6	0.02	0.262	0.002	0.017	0.044	<0.0005
MSA-C	<0.0005	<0.0005	<0.005	<0.0005	<0.0005	0.002

Comparison of MRS ratios in the cerebellum between patients with SCA or MSA-C and the healthy controls.

### Patients with SCA

Significantly lower NAA/Cr in the cerebellar hemispheres and vermis in all patients with SCA (SCA2, SCA3 or SCA6) (*p*<0.0005∼0.017) and lower Cho/Cr in the cerebellar hemispheres and vermis in patients with SCA2 (*p*<0.0005) were found. Significantly lower NAA/Cho in the cerebellar hemispheres and vermis were also noticed in almost all patients with SCA *(p<0.0005∼0.002)*, except for NAA/Cho in the vermis in patients with SCA2 (*p* = 0.077).

#### Comparison between SCA and MSA-C

NAA/Cr, Cho/Cr and NAA/Cho in the cerebellar hemispheres and vermis were significantly lower in patients with MSA-C, compared with those with SCA3, except for the NAA/Cho in the vermis (*p* = 0.626). Significantly lower NAA/Cr, Cho/Cr and NAA/Cho were found in the patients with MSA-C than those with SCA6, except for NAA/Cho in the cerebellar hemispheres (*p = 0.086*) and NAA/Cr in the vermis (*p = 0.185*). There was no significant difference between patients with SCA 2 or MSA-C. (Table 5)

**Table 5:** The *p*-values derived from statistical analyses in early stage of diseases when SARA scores were below 10 unit points.

	Cerebellar hemispheres			Cerebellar vermis		
	NAA/Cr	Cho/Cr	NAA/Cho	NAA/Cr	Cho/Cr	NAA/Cho
SCA2	0.215	0.802	0.072	0.242	0.212	0.639
SCA3	<0.0005	<0.0005	0.001	<0.0005	<0.0005	0.626
SCA6	0.001	<0.0005	0.086	0.185	0.002	0.002

Comparison of MRS ratios in the cerebellum between patients with subtypes of SCA and MSA-C.

#### Comparison between subtypes of SCAs

Patients with SCA2 had a lower NAA/Cr and Cho/Cr in the cerebellar hemispheres and vermis than those with SCA3 or SCA6. However, there were no significant difference in NAA/Cho between patients with SCA2, SCA3 or SCA6, except for lower NAA/Cho in the vermis in patients with SCA6, as compared with those with SCA2 (*p* = 0.038) or SCA3 (*p* = 0.005). (Table 6)

**Table 6:** The *p*-values derived from statistical analyses in early stage of diseases when SARA scores were below 10 unit points.

	SCA2 vs. SCA3	SCA2 vs. SCA6	SCA3 vs. SCA6
Hemispheric NAA/Cr	<0.0005	0.016	0.531
Hemispheric Cho/Cr	<0.0005	<0.0005	0.708
Hemispheric NAA/Cho	0.876	0.851	0.791
Vermis NAA/Cr	0.004	0.476	0.162
Vermis Cho/Cr	0.027	0.019	0.066
Vermis NAA/Cho	0.689	0.038	0.005

Comparison of MRS ratios in the cerebellum between subgroups of SCAs.

#### Correlations between clinical severity of ataxia and MRS ratios

The SARA scores significantly correlated with the disease duration (*p*<0.0005) and MRS ratios, including NAA/Cr and Cho/Cr in the cerebellar hemispheres, NAA/Cr and Cho/Cr in the vermis (*p*<0.0005), NAA/Cho in the cerebellar hemispheres (*p* = 0.037) and NAA/Cho in the vermis (*p = 0.046*). When SARA scores were analyzed alongside the MRS data, an inverse correlation was found between MRS ratios and SARA scores in all subtypes of SCA and MSA-C. The lower the metabolite ratios, the higher the SARA scores.

NAA/Cho correlated well with the duration of disease (*p* = 0.037 in the cerebellar hemispheres; *p* = 0.006 in the vermis). However, the disease duration did not correlate with NAA/Cr *(p = 0.254)* or Cho/Cr (*p* = 0.354) in the cerebellar hemispheres, or NAA/Cr *(p = 0.394)* or Cho/Cr (*p* = 0.251) in the vermis.

## Discussion

SCA and MSA-C share many clinical features but have distinct underlying pathophysiologies leading to progressive neuronal dysfunction. In this study, proton MRS was used to monitor the neuronal damage *in vivo* and to explore the differences between different subtypes of SCA and MSA-C. The NAA/Cr was particularly lower in the cerebellar hemispheres and vermis in patients with MSA-C or SCA2 than those with SCA3 or SCA6.

Previously, lower NAA/Cr had been reported in patient with SCA1, SCA2, SCA6 or MSA-C. [14]–[17]. Of note is that, patients with SCA2 had lower NAA/Cr than those with SCA6, even they were of similar clinical severity. [15], [16] Hadjivassiliou M et al. [21]had demonstrated a significant atrophy and lower NAA/Cr in both vermis and cerebellar hemispheres in patients with SCA6. SCA6 is a disease with relatively pure and slowly progressive ataxia and a late age at onset. The less reduction of NAA/Cr in SCA6 may reflect a better reserved functioning of surviving neurons.

Lower Cho/Cr in the cerebellar hemispheres and vermis was found in patients with SCA2, MSA, SCA3 and SCA6. In particular, lower Cho/Cr in the cerebellar hemispheres and vermis in patients with MSA-C than those with SCA2 was noticeable. Although the patient numbers were limited in SCA1 and SCA17 groups, the NAA/Cr and Cho/Cr in the cerebellar hemispheres in SCA1 were lower than those with SCA3 or SCA6 but higher than those with SCA2 and MSA-C.

Therefore, MRS features suggest that the neuronal integrity and the activities of membrane synthetic enzymes are better preserved in patients with SCA3, SCA6, followed by SCA17, SCA1 and worst in patients with SCA 2 or MSA-C. The activities of membrane enzyme synthesis are even worse in MSA-C than SCA2.

### NAA/Cho

NAA/Cho has been widely used as a marker for cerebral metabolism [8], [12], [22], [23]. In this study, we observed significantly lower NAA/Cho in the cerebellar hemispheres in all patients with SCA or MSA-C, except for SCA 17. Patients with SCA2 had a lower NAA/Cho in the cerebellar hemispheres, reflecting a more severe cerebellar damage. The NAA/Cho features indicate that cerebellar metabolisms were higher in SCA3, SCA6 and less so in patients with SCA 2 or MSA-C, consistent with previous PET reports [24].

### Observations in the early sages of the diseases

In the early stages, when cerebellar signs were not salient, the decrease in NAA/Cr, Cho/Cr and NAA/Cho in patients with MSA-C or SCA has already been noticeable. Therefore, measurement of neurochemical changes by MRS seems to be superior to clinical observation.

The Cho/Cr in the vermis in patients with SCA6 was higher than those with SCA3, SCA2 and even the healthy controls. (Table 3) This might be attributable to the limited size (only 4 patients with SCA6) of the patients in the early stage and might not be representative. Lower NAA/Cr in the cerebellar hemispheres and vermis in patients with MSA-C might implicate that a significant and faster cerebellar pathology occurring very early in MSA-C [19].

SCA2 and MSA-C are two most severe ataxia diseases with similarly rapid reductions in NAA/Cr, Cho/Cr and NAA/Cho. Previous studies [14]–[16] suggested that lactate peak and myo-inositol levels might be used to differentiate SCA2 from MSA-C. We found that, Cho/Cr in the vermis and NAA/Cho in the cerebellar hemispheres were lower in patients with MSA-C than those with SCA2 in the early stage of the diseases, (Table 3) and, as time went by, patients with MSA-C tended to have lower Cho/Cr than those with SCA2, despite that their NAA/Cr was similar. (Table 2) This trend may reflect the initial and early neuronal or axonal damage, followed by progressive and rapid impairment of membrane activities specifically in MSA-C. Thus, the Cho/Cr may be used as another marker to differentiate MSA-C from SCA2.

Pathologically, SCA2 is characterized by a marked loss of Purkinje cells in the cerebellar cortex, as well as a loss of myelinated fibers with gliosis in the inferior and middle cerebellar peduncles, cerebellar white matter and fasciculus cuneatus [25], [26]. In SCA3, the middle cerebellar peduncle and the central white matter of the cerebellum are heavily involved, while the cerebellar cortex and Purkinje cells are mostly spared [27]. In SCA6, the pathology occurs mainly in the Purkinje cells of the cerebellar cortex, leaving the dentate nucleus, deep cerebellar white matter and cerebellar peduncles unaffected [28], [29]. The results in this MRS study correlate well with these differences in neuropathology. The synaptic loss in cerebellum and brainstem is most severe in SCA2, followed by SCA1 and SCA6 [30], [31]. The changes in MRS are similarly ranked, as reported by Oz G et al. using 4T MRS [32].

### Clinical significance

Differentiation of subtypes of ataxias was once considered possible using NAA, myo-inositol, total Cr, glutamate and glutamine with a high field 4T MR spectroscopy [16], which, however, is not readily accessible in most clinical settings. In contrast, in this study, we demonstrated that subtle differences between subtypes of SCAs and MSA-C could be discerned simply by using NAA/Cr, Cho/Cr and NAA/Cho obtained from a 1.5 T MRI. Furthermore, MRS features correlate well with clinical SARA scores. Therefore, MRS biomarkers can be utilized to noninvasively assess neuronal and glial status in individual patients with ataxia.

One limitation of this study is the small sample sizes. Although the overall sample sizes are impressive, fewer than 10 subjects were recruited for the SCA1 and SCA17 groups. The limited sample size is difficult to avoid and complicates the statistical analyses, interpretation and inferences at the sub-group level. Since SCA1, 2, 3, 6 and 17 are all polyglutamine diseases with unstable CAG repeat expansion, it would be conceivable to correlate the CAG numbers with clinical and MRS features and to prospectively compare the progressive changes in different subtypes of SCA in a longitudinal study in the future.

### Conclusion

By using MRS, significantly lower MRS ratios, suggestive of neuronal dysfunction and membrane activities impairment, which correlate well with clinical deterioration, were found in patients with SCAs and MSA-C. MRS may be used to *in vivo* evaluate and monitor the progression of neurodegeneration in ataxia, potentially even in the early stages of SCA or MSA-C.
